# Epidemiology and Molecular Characteristics of *mcr-9* in *Citrobacter* spp. from Healthy Individuals and Patients in China

**DOI:** 10.1128/spectrum.01346-22

**Published:** 2022-11-14

**Authors:** Xiaoyang Ju, Siheng Wang, Xuemei Yang, Weihua Han, Chang Cai, Yuchen Wu, Congcong Liu, Jiao Qian, Xiaofei Zhao, Xiang Qian, Qiaoling Sun, Rong Zhang, Gongxiang Chen

**Affiliations:** a Clinical Microbiology Laboratory, The Second Affiliated Hospital of Zhejiang University School of Medicine, Zhejiang University, Hangzhou, China; b Department of Infectious Diseases and Public Health, Jockey Club College of Veterinary Medicine and Life Sciences, City University of Hong Kong, Hong Kong, China; c China Australia Joint Laboratory for Animal Health Big Data Analytics, College of Animal Science and Technology, Zhejiang Agricultural and Forestry University, Hangzhou, China; d Clinical Laboratory Department, Taizhou Hospital of Zhejiang Province, Wenzhou Medical University, Taizhou, China; e The Affiliated Hospital of Hangzhou Normal University, Hangzhou, China; f Department of Clinical Laboratory, Hangzhou Hospital of Traditional Chinese Medicine, Hangzhou, Zhejiang, China; g Department of Laboratory Medicine, The First Affiliated Hospital, Zhejiang University School of Medicine, Hangzhou, Zhejiang, China; University of California, Davis

**Keywords:** *mcr-9*, *Citrobacter* spp., colistin, plasmid, *Citrobacter freundii*

## Abstract

With the globally prevailing carbapenemase-producing (CP) *Citrobacter* spp., polymyxin antibiotics have been reconsidered as one of the last-resort treatment options. Our study was conducted to investigate the prevalence of *mcr-9* in *Citrobacter* species. From October to November 2021, 650 fecal samples and 215 *Citrobacter* isolates were collected from healthy individuals and infected patients, respectively. Isolates were screened for the presence of the *mcr-9* gene by the PCR method. *mcr-9*-carrying strains were identified by matrix-assisted laser desorption ionization–time of flight (MALDI-TOF) mass spectrometry. Due to the susceptibility to colistin, *Citrobacter* spp. isolates were first induced to increase the expression of *mcr-9* on China blue agar plates containing colistin and were then subjected to conjugation experiments. Whole-genome sequencing was performed on the Illumina NovaSeq PE150 system. The prevalence of *mcr-9* in the *Citrobacter* genus from healthy guts and infected patients was 0.62% and 1.86%, respectively. In all *mcr-9*-positive strains, MICs of polymyxin B were observed at ≤2 μg/mL, displaying a nonresistant phenotype. As for conjugation experiments, only one isolate successfully transferred the *mcr-9* gene to Escherichia coli C600. Whole-genome sequencing showed that eight *mcr-9*-positive *Citrobacter* isolates carried *mcr-9* and genes encoding resistance to beta-lactam antibiotics, including *bla*_CMY_, *bla*_DHA_, *bla*_SHV_, *bla*_TEM_, and *bla*_CTX-M_. We also discovered that *mcr-9* could be located on the pKPC-CAV1321 plasmid. Our study investigated the prevalence of *mcr*-*9* in *Citrobacter* spp. in both healthy individuals and infected patients and described the carriage of *mcr-9* on the pKPC-CAV1321 plasmid for the first time.

**IMPORTANCE** The emergence of *mcr* homologues posed a serious threat to the therapeutic efficiency of polymyxin antibiotics. Citrobacter freundii is generally regarded as an opportunistic pathogen associated with a variety of nosocomial infections. In this study, we investigated the prevalence of *mcr-9* in *Citrobacter* spp. isolates from healthy individuals and infected patients and highlighted the importance of the rational use of antibiotics. In addition, this epidemiological investigation is the first to describe the carriage of *mcr-9* on plasmid pKPC-CAV1321 and confirms the horizontal transfer of this plasmid. Our research may shed new light on further studies of *mcr-9* dissemination in humans.

## INTRODUCTION

The genus *Citrobacter*, a member of the *Enterobacterales* family, is commonly found in water, soil, and the guts of humans and animals. Previous studies have shown that Citrobacter freundii, as the representative within *Citrobacter* species, is emerging as the cause of a variety of opportunistic infections involving urinary tract, respiratory tract, and wound infections (1–3). Recently, the escalating increase in multidrug-resistant (MDR) strains, particularly carbapenemase-producing (CP) C. freundii, poses a serious threat to public health on an international scale (4, 5). Due to the lack of effective antibiotics, polymyxin (colistin and polymyxin B), a neglected antibiotic, returned to the spotlight as one of the last resorts against serious infections caused by MDR strains (6).

In 2016, *mcr-1*, the first mobile colistin resistance gene was first reported in humans and food animals (7). It not only presented an enormous challenge to the therapeutic efficiency of colistin but also caused a global panic over antibiotic barrenness (8). To date, 10 plasmid-borne *mcr* homologues (*mcr-1* to *mcr-10*) have been detected in multitudinous genera of *Enterobacterales* from animals, humans, and the environment (9, 10). Among them, *mcr-9* is the second most widely spread gene, following *mcr*-*1*, and has been identified in 40 countries across six continents (11).

The human gut is considered as a reservoir of resistance genes and acts an important role in horizontal gene transfer. According to the reports from Wang et al., the prevalence of *mcr*-*1*-positive Escherichia coli in healthy individuals was as high as 14.3% in 2016. Since China banned colistin as an animal feed additive in 2017, the prevalence of *mcr*-*1* has displayed a marked decline (*P < *0.0001) (12).

Considering the importance of *mcr* genes, we carried out the *mcr*-*9* screening in fecal samples and clinical isolates from healthy individuals and patients, respectively, to investigate its prevalence and gain an insight into the microbiological features of *mcr-9*-carrying *Citrobacter* spp. from both healthy individuals and patients.

## RESULTS

### Prevalence of *mcr-9*-carrying *Citrobacter* spp.

A total of eight *mcr-9*-positive strains were initially identified as C. freundii by matrix-assisted laser desorption ionization–time of flight (MALDI-TOF), but two of strains were further confirmed to be Citrobacter portucalensis using average nucleotide identity(ANI). Three C. freundii strains and one *C. portucalensis* strain were from healthy individuals, and the others were from infected patients (Table 1). Of the 215 *Citrobacter* spp. isolated from the infection samples, 4 (1.86%) were confirmed to carry *mcr*-9, which was higher than the prevalence of *mcr-9*-carrying *Citrobacter* spp. from healthy guts (0.62%, 4/650), but no significant difference was observed via Chi-square test (*P = *0.214, >0.05).

**TABLE 1:** Prevalence of *mcr*-*9*-positive *Citrobacter* spp. from patients and healthy individuals^*a*^

Group	Total	*mcr-9* positive (%)	*mcr-9* negative (%)
Clinical isolates	215	4 (1.86)	214 (98.14)
Healthy samples	650	4 (0.62)	646 (99.38)

a χ^2^ = 1.543; *P* = 0.214.

### Antimicrobial susceptibility testing and conjugation experiments.

According to CLSI breakpoints, all *Citrobacter* spp. isolates verified to carry *mcr*-*9* are nonresistant to polymyxin B (Table 2), and the MICs of these strains were <2 μg/mL. None of the isolates were resistant to carbapenem antibiotics, including imipenem, meropenem, and ertapenem. The conjugation experiments were carried out for eight *mcr-9*-positive *Citrobacter* spp. isolates, while only one isolate, number 156, recovered from healthy individuals, successfully delivered the *mcr-9* to E. coli EC600.

**TABLE 2:** Antimicrobial susceptibility testing results of eight *mcr-9*-positive *Citrobacter* spp.^*a*^

Group	Strain	MIC (μg/mL) for:														
		IPM	MEM	ETP	CMZ	CAZ	CTX	TZP	SCF	CAV	FEP	PB	TGC	CIP	AK	ATM
Clinical isolates	ZY-5	≤1	≤1	≤2	128	>128	16	16/4	≤8/4	≤0.5/4	≤4	≤0.5	4	16	8	128
	wm52	≤1	≤1	≤2	32	64	128	≤8/4	≤8/4	≤0.5/4	≤4	1	0.5	≤1	8	64
	21435	≤1	≤1	≤2	16	≤2	≤4	≤8/4	≤8/4	≤0.5/4	≤4	≤0.5	1	>32	≤4	≤4
	F1-34	≤1	≤1	≤2	32	>128	32	64/4	16/8	≤0.5/4	≤4	≤0.5	1	≤1	≤4	32
Healthy human intestinal isolates	56	≤1	≤1	≤2	64	≤2	8	≤8/4	≤8/4	≤0.5/4	≤4	1	0.5	≤1	8	≤4
	82	≤1	≤1	≤2	16	≤2	≤4	≤8/4	≤8/4	≤0.5/4	≤4	≤0.5	1	≤1	8	≤4
	146	≤1	≤1	≤2	32	≤2	≤4	≤8/4	≤8/4	≤0.5/4	≤4	1	0.5	≤1	≤4	≤4
	156	≤1	≤1	≤2	32	≤2	16	≤8/4	≤8/4	≤0.5/4	≤4	≤0.5	≤0.25	≤1	≤4	≤4

a IPM, imipenem; MEM, meropenem; ETP, ertapenem; CMZ, cefmetazole; CAZ, ceftazidime; CTX, cefotaxime; TZP, piperacillin/tazobactam; SCF, cefoperazone/sulbactam; CAV, ceftazidime/avibactam; FEP, cefepime; PB, polymyxin B; TGC, tigecycline; CIP, ciprofloxacin; AK, amikacin; ATM, aztreonam.

### Genetic analysis of *mcr-9*-positive *Citrobacter* spp.

The genetic characteristics of the eight *mcr-9*-positive isolates are presented in Table 3. Compared with isolates from healthy people, *Citrobacter* spp. from infected patients presented more abundant resistance genes and plasmid types. All of the eight strains were found to carry *mcr-9* and genes encoding resistance to beta-lactam antibiotics, including *bla*_CMY_, *bla*_DHA_, *bla*_SHV_, *bla*_TEM_, and *bla*_CTX-M_. In addition, aminoglycoside (*aac*) and sulfonamide (*sul1*) resistance genes were detected in all four *Citrobacter* spp. strains from patients.

**TABLE 3:** Antibiotic resistance genes and plasmid replicon typing of eight isolates of *mcr-9*-carrying *Citrobacter* spp.

Group	Strain	Species	Resistance gene	Plasmid
Clinical isolates	ZY-5	C. freundii	*aac(6′)-Ib3*, *aac(6′)-Ib-cr*, *aac(6′)-IIc*, *bla*_SHV-12_, *catA2*, *bla*_DHA-1_, *bla*_CMY-135_, *bla*_TEM-1B_, *qnrB4*, *mcr-9*, *sul1*, *dfrA19*, *tet*(D)	IncFII(SARC14), IncHI2, IncHI2A
	wm52	C. portucalensis	*aac(6′)-Ib3*, *aac(6′)-Ib-cr*, *ant(2″)-Ia*, *bla*_SHV-12_, *bla*_CTX-M-9_, *catA1*, *bla*_CMY-125_, *qnrB9*, *mcr-9*, *sul1*, *dfrA16*, *tet*(A)	IncFIB(K), IncHI2, IncHI2A
	21435	C.freundii	*aac(6′)-Ib-cr*, *bla*_CMY-117_, *bla*_TEM-1B_, *qnrB6*, *mcr-9*, *sul1*, *dfrA17*, *dfrA27*	IncFIB(pHCM2), IncHI2, IncHI2A
	F1-34	C.freundii	*aac(6′)-If*, *cmlA1*, *bla*_CMY-79_, *bla*_CMY-116_, *mcr-9*, *sul1*, *fosA3*	Col(IMGS31), pKP1433, pKPC-CAV1321
Healthy human intestinal isolates	56	C. portucalensis	*bla*_CMY-2_, *qnrB9*, *mcr-9*, *sul1*	
	82	C.freundii	*bla*_CMY-78_, *mcr-9*	pKPC-CAV1321
	146	C.freundii	*aph(6)-Id*, *bla*_CMY-116_, *bla*_CMY-79_, *mcr-9*	IncFIB(pECLA), pKPC-CAV1321, repA(p447)
	156	C.freundii	*bla*_CMY-48_, *qnrB38*, *qnrB60*, *mcr-9*	pKPC-CAV1321

Furthermore, *mcr-9*-positive *Citrobacter* spp. isolated from sites of infection, except for one isolate, carried IncHI2 and IncHI2A replicons, among which IncHI2 is the predominant replicon type reported to carry *mcr*-*9* (13). The exception was isolate F1-34, which contained the pKPC-CAV1321 type plasmid rather than IncHI2 or IncHI2A. Surprisingly, the majority of *Citrobacter* spp. isolated from healthy guts appeared to carry the pKPC-CAV1321 plasmid but not IncHI2 or IncHI2A. In addition, no plasmid was observed in isolate number 56.

## DISCUSSION

In this study, we describe the prevalence of *mcr-9* in *Citrobacter* spp. from both healthy individuals and infected patients. Several scattered *mcr-9*-carrying C. freundii isolates were reported in animal and patient samples in previous studies (14–16), but information regarding clinical epidemiology and mechanisms of resistance to colistin in *Citrobacter* genera is lacking. According NCBI databases (https://www.ncbi.nlm.nih.gov/pathogens/isolates/#AMR_genotypes:(mcr-9*)), isolates of C. freundii carrying *mcr-9* have been detected in 12 countries across 4 continents as of March 2022, including two isolates in China. However, no reports of the *mcr-9* gene located in *C. portucalensis* were reported. *C. portucalensis* is a novel species of the genus *Citrobacter* that was closely related to C. freundii and first isolated from an aquatic sample in Portugal in 2017 (17).

It is of great significance to detect *Citrobacter* isolates carrying *mcr-9* in both healthy people and infected patients. In recent years, MDR *Citrobacter* spp. attracted increasing attention, since infections caused by these bacteria were always life-threatening and difficult to treat. What’s more, C. freundii is a commensal of the intestinal tract of humans and animals and plays an important role in carrying and transferring various resistance genes.

Although the majority of previously reported *mcr-9*-carrying isolates, including eight *Citrobacter* spp. isolates in this study, were not resistant to colistin (18), it has been proved that the expression of *mcr-9* could be inducible by subinhibitory concentrations of colistin in the presence of *qseB* and *qseC* genes, the MIC levels were therefore increased (19). This suggests that the clinical use of colistin may induce resistance to colistin in *mcr*-*9*-positive isolates and accelerate the dissemination of *mcr*-*9* among potential pathogens. Subsequently, the emergence of colistin-resistant strains will further limit the clinical antibiotic options, resulting in more serious global resistance. It is suggested that rational use of antibiotics is crucial to reduce microbial resistance.

IncHI2 plasmids were the predominant replicon type carrying *mcr*-*9* (13) and could increase the dissemination of *mcr*-*9* in carbapenem-resistant *Enterobacteriaceae* (CRE) (20). Surprisingly, our data suggested that *mcr-9* can also be located on the type of pKPC-CAV1321 plasmid. To the best of our knowledge, our study is the first report of such a gene on plasmid pKPC-CAV1321. As described above, only isolate 156 successfully transmitted the *mcr*-*9* gene to E. coli EC600 via conjugation experiment, and the conjugant was also tested by whole-genome sequencing. The WGS analysis of the conjugant indicated that the *mcr*-*9* gene of isolate 156 was indeed located on the pKPC-CAV1321 plasmid. Our finding suggested that *mcr*-*9* can be transferred into other microbial pathogens along with this plasmid, thereby accelerating the spread of *mcr*-*9*.

In addition, analysis of the plasmids showed that *mcr*-*9* spread in *Citrobacter* spp. may be related to the antibiotic environment where the isolates were grown. For clinical isolates, transmission of *mcr*-*9* is largely by means of IncHI2 and IncHI2A among multidrug-resistant pathogens, as these superplasmids may carry a large number of resistance genes required for survival. However, spread of *mcr*-*9* in *Citrobacter* spp. among healthy person possibly was connected with plasmid pKPC-CAV1321.

Our study investigates the prevalence of *mcr*-*9* in the genus *Citrobacter* from both healthy individuals and patients and reports the carriage of *mcr-9* on plasmid pKPC-CAV1321 for the first time.

## MATERIALS AND METHODS

### Sample collection.

From October to November 2021, a total of 650 fecal samples were collected from healthy individuals. Fecal samples were collected from subjects who underwent routine physical examinations within 3 days, excluding those with gastroenteritis or chronic diseases. A total of 215 *Citrobacter* isolates were collected from infected patients. Infected patients were those who had been diagnosed by a doctor with a bacterial infection in at least one body system or region, including sputum, secretions, urine, blood, feces, and tissues.

After being enriched in LB broth at 37°C for 6 to 8 h, all fecal samples were screened for the *mcr-9* gene by PCR using previously published primers (21), and the clinical isolates obtained from infected patients were directly screened for the *mcr-9* gene using the PCR method. PCR-positive samples were further purified and subjected to verification of *mcr-9* by Sanger sequencing. Next, matrix-assisted laser desorption ionization–time of flight (MALDI-TOF; Bruker, Germany) was performed to confirm the identification of *mcr-9*-carrying isolates.

### Antimicrobial susceptibility testing.

The broth microdilution method was used to examine the sensitivity of *mcr-9*-positive isolates to common antibiotics, including imipenem, meropenem, ertapenem, cefmetazole, ceftazidime, cefotaxime, piperacillin/tazobactam, cefoperazone/sulbactam, ceftazidime-avibactam, cefepime, polymyxin B, tigecycline, ciprofloxacin, amikacin, and aztreonam. The MICs of most antimicrobial agents, with the exception of tigecycline, were interpreted according to Clinical and Laboratory Standards Institute (CLSI) guidelines (22). Results of tigecycline were judged with reference to the breakpoints of the European Committee for Antimicrobial Susceptibility Testing (EUCAST) (https://www.eucast.org/). Based on the MIC interpretation criteria of the CLSI guidelines, isolates with a MIC of ≤2 μg/mL were classified as polymyxin B intermediate and those with a MIC of ≥4 μg/mL as polymyxin B resistant.

### Conjugation experiments.

Considering that *mcr*-*9*-positive isolates showed a nonresistant phenotype but could be induced in the colistin-containing plate (19), we induced the expression of *mcr*-*9* by subculturing successive generations onto a China blue agar plate containing colistin to increase the MIC levels. Conjugation experiments were carried out between *mcr-9*-carring *Citrobacter* spp. isolates, the MICs of which were induced to 2 μg/mL, and rifampin-resistant Escherichia coli C600. The donor was a *Citrobacter* spp. with EC600 as a recipient. Conjugants were selected on Mueller-Hinton (MH) agar plates supplemented with 600 μg/mL rifampicin and 1.5 μg/mL colistin.

### Whole-genome sequencing.

Genomic DNA was separated from *mcr*-*9*-positive isolates with the PureLink genomic DNA minikit (Invitrogen, USA), following the instructions provided, and sequenced on the Illumina NovaSeq PE150 system by Novogene, China. The short-read data were assembled using SPAdes v3.15.4 (http://cab.spbu.ru/software/spades/). Identification of antibiotic resistance genes and plasmid replicon typing were conducted using the Center for Genomic Epidemiology website (http://www.genomicepidemiology.org/).

### Data availability.

The data sets presented in this study can be found under BioProject number in online repositories. The names of the repository/repositories and accession numbers can be found below: PRJNA827125.
